# The efficacy of 40 mg versus dose de-escalation to less than 40 mg of afatinib (Giotrif) as the first-line therapy for patients with primary lung adenocarcinoma harboring favorable epidermal growth factor mutations

**DOI:** 10.18632/oncotarget.18746

**Published:** 2017-06-27

**Authors:** Chien-Ying Liu, Chih-Liang Wang, Shih-Hong Li, Ping-Chih Hsu, Chih-Hung Chen, Ting-Yu Lin, Chih-Hsi Kuo, Yueh-Fu Fang, How-Wen Ko, Chih-Teng Yu, Tai-Yun Yang, Cheng-Ta Yang

**Affiliations:** ^1^ Division of Pulmonary Oncology, Department of Thoracic Medicine, Chang Gung Memorial Hospital, Taipei, Taiwan; ^2^ School of Medicine, Chang Gung University, Taoyuan, Taiwan

**Keywords:** afatinib, dose de-escalation, epidermal growth factor mutations, lung adenocarcinoma, therapeutic efficacy

## Abstract

The choice of a first-line therapy for lung cancer is a crucial decision that can impact the survival as well as the quality of life of a patient. Inhibitors of epidermal growth factor receptor (EGFR) such as afatinib, erlotinib, and gefitinib have previously been used to treat non-small cell lung cancer harboring favorable EGFR mutations. Although afatinib has greater efficacy than other EGFR inhibitors, adverse events related to its use can result in the discontinuation of the therapy. In this study, we compared the therapeutic efficacy in lung cancer patients of a regimen of 40 mg/day of afatinib with that of a lower dose regimen of <40 mg/day resulting either from a lower starting dose of 30 mg/day or dose adjustment. Seventy-nine patients were treated with 40 mg/day and 67 received de-escalated doses of <40 mg/day. There was no significant difference in the clinical characteristics of the two groups except that the proportion of patients with a body weight of 50 kg or more was greater in the 40 mg/day group. Otherwise, there were no significant differences between the two groups in the average time to treatment failure (TTF), the rates at which the administration of a second-line therapy was necessary, or the frequency and severity of adverse events. Overall, these results suggest that it is possible to calibrate the dosage of afatinib to suit individual patient parameters such as low body weight, and that such calibration can be advised based on the given patient’s individual experience of the drug.

## INTRODUCTION

According to estimates from Globocan, lung cancers accounted for 12.9 percent of all new cancer cases worldwide in 2012, with the highest incidence and mortality rates occurring in China and the Western Pacific region, and incidence rates being higher in general in the developing world [1]. Lung cancers also have a high fatality (incidence to mortality) ratio of 0.87, resulting in the availability of only a very small window of time for effective treatment. Non-small cell lung cancer (NSCLC) accounts for 85% of all lung cancers, and has a low 5-year survival rate of only 15% [2]. The treatment of NSCLC has been attempted with standard chemotherapeutic agents like cisplatin, as well as with new drugs such as epidermal growth factor receptor tyrosine kinase inhibitors (EGFR-TKIs). The latter drugs have been used because it is the occurrence of mutations in the EGFR tyrosine kinase domains that activates Ras-mediated anti-apoptotic pathways in lung cells, leading to uncontrolled proliferation [3].

Worldwide, the incidence of lung cancers and adenocarcinomas of the lung (a subset of NSCLC) is highest in China and in the Western Pacific region. Cigarette smoking is a contributory factor; however, adenocarcinomas have also been found in individuals who have never smoked. Individuals of Asian ancestry and men are particularly prone to suffering from NSCLC [2]. Several phase III studies have confirmed that patients with advanced lung cancer and EGFR mutations benefit more from first-line targeted EGFR-TKI therapies, such as gefitinib and erlotinib, than from the standard platinum doublet chemotherapy [4]. The IPASS study, a large-scale, randomized, clinical trial involving 1200 Asian NSCLC patients, was conducted in order to compare the efficacy of an EGFR-TKI (gefitinib, in this case) with a standard chemotherapy (carboplatin-paclitaxel) [4, 5]. Gefitinib therapy was found to have a lower hazard ratio (HR) of 0.48 in patients with tumors resulting from deletions in exon 19 and an L858R substitution in exon 21 of the EGFR. Interestingly, this study also highlighted the lower efficacy of gefitinib compared with chemotherapy in NSCLC patients without these characteristic mutations. Diagnostic tests based on the EGFR-TKI mutations to identify exon 19 deletion and L858R mutations have therefore become an integral component of treatment protocols for NSCLC. Gefitinib is a reversible inhibitor of EGFR-TK activity and is therefore administered in high dosages (250 mg/day); these high dosages result, in turn, in adverse events like diarrhea and skin rashes. In contrast, afatinib, an irreversible inhibitor, has been shown to have better anti-tumor activity at lower dosages of only 40-50 mg/day [6, 7]. The most significant difference between the two inhibitors is the fact that, for NSCLC patients, treatment with afatinib increases the duration of progression-free survival (PFS) in comparison with gefitinib (median 11.0 months versus median 10.9 months, HR: 0.73, *p*=0.017), as well as the time to treatment failure (TTF) (median 13.7 months versus median 11.5 months, HR: 0.73, *p*=0.0073) [6].

Nonetheless, significantly higher rates of adverse events, including diarrhea and acneiform skin rashes (grade 3 and grade 4 events), are more associated with afatinib therapy than with gefitinib therapy. Relatedly, while afatinib is now considered a first-line therapy for NSCLC harboring favorable EGFR mutations, adverse events resulting from this drug sometimes require discontinuation of the therapy.

In this study, therefore, we examined the efficacy of a labeled dose (40 mg/day), as well as a lower starting dose of 30 mg/day or downward dose adjustment from 40 mg/day during the treatment period (collectively described as <40 mg/day), of afatinib as a first-line therapy for NSCLC patients bearing favorable EGFR-TK mutations. The goal was, in part, to better understand whether factors such as low lean body mass can be considered in calibrating afatinib doses in order to reduce the severity of adverse events while still achieving therapeutic effectiveness.

## RESULTS

A total of 146 patients who received afatinib therapy from May 2014 (2014/05/05) to December 2015 (2015/12/14) were included in the analysis. Seventy-nine patients were included in the 40 mg/day group and 67 patients were included in the <40 mg/day group. The adjustment of dosage to 30 mg was done as per the incidence and tolerance of adverse events. The baseline characteristics of the patients in both treatment groups are listed in Table 1. The mean age of the patients in the 40 mg/day group was 62.4 ± 11.0 years while that of the patients in the <40 mg/day group was 64.3 ± 11.7 years, meaning that there was no significant difference in age between the two groups (p=0.4276). The proportion of female patients in the <40 mg/day group (64.2%) was greater than that in the 40 mg/day group (44.3%) (p=0.02), while the proportion of patients with a body weight of 50 kg or more was greater in the 40 mg/day group (odds ratio, OR: 2.337, 95% confidence interval, 95% CI: 1.067–2.622, p=0.0400, compared with the <40 mg/day group). This difference in the proportion of patients with a body weight of 50 kg or more in the two treatment groups in this study, which was consistent with the bias towards inclusion of female patients in the lower dose group, was significant. (50 kg was chosen as the cut-off value representing low body weight on the basis of analyses conducted by previous LUX-Lung 3, LUX-Lung 6, and LUX-Lung 7 trials and with reference to data regarding the average body weights of both male and female Taiwanese adults from Taiwan’s Ministry of Health and Welfare [8].) The Eastern Cooperative Oncology Group performance status (ECOG PS) scores were low for most of the participating patients, suggesting that they were generally ambulatory and able to perform most daily tasks. This may have been in part because patient inclusion in this study was biased towards lower ECOG PS scores owing to insurance compliance norms. The inclusion of female patients in the lower dosage group was also influenced by the fact that patients with lower BMI and body weight are less likely to tolerate the adverse effects of afatinib [9]. The distribution of exon 19 deletion mutations as well as exon 21 L858R mutations in each dosage group was unbiased, as can be seen from the relevant p-values listed in Table 1.

**Table 1:** Baseline demographic and disease characteristics of patients

Characteristics	Total	First-line treatment with afatinib		*p* value
		40 mg/day	< 40 mg/day	
Patients, No.	146	79	67	
Male/female	68 / 78	44 / 35	24 / 43	0.0200
Age (mean±SD)	63.2 ± 11.3	62.4 ± 11.0	64.3 ± 11.7	0.4276
Body mass index (BMI)	23.8 ± 4.0	23.6 ± 3.7	24.0 ± 4.4	0.7347
Body weight (No., ≧50 / <50 kg)	116/ 30	68 /11	48 / 19	0.0400
Smoking status				
Never smokers/smokers (Former+ Current)	109 / 37	55 / 24	54 / 13	0.1810
Stage of disease				
IIIB/IV	16 / 130	12 / 67	4 / 63	0.1098
ECOG PS prior to therapy				
0-1/≧2	123 / 23	70 / 9	53 / 14	0.2645
EGFR mutation				0.5599
Exon 19 deletion	73	42	31	0.5066
Exon 21 L858R	61	32	29	0.7398
Others *	12	5	7	0.9586
Pleural effusion	53	23	30	0.0586
Brain metastasis	29	13	16	0.3015
Bone metastasis	71	40	31	1.0000
Liver	14	7	7	0.7840
Adrenal gland	6	6	1	0.1407
Local radiotherapy	40	20	20	0.2499
Brain	15	7	8	0.5924
Bone	22	10	12	0.4871
Other lesions**	3	3	0	0.2499

No., number

*In 40 mg/day of afatinib group, one patient had an L861Q EGFR mutation, and four had exon 20 insertions. In the <40 mg/day of afatinib group, one patient had a G719A EGFR mutation, two had a G719S mutation, one had a G719S mutation and an exon 20 insertion, one had an L861Q EGFR mutation, and two had exon 20 insertions.

**In the 40 mg/day of afatinib group, three patients had radiation therapy for metastatic lung tumors.

In this study, two patients receiving the 40 mg/day regimen and one patient receiving the <40 mg/day regimen exhibited a complete response to the given treatment (Table 2). The rates of partial response to treatment were also similar in both dosage regimen groups. The objective response rates were 72.2% and 71.6% in the 40 mg/day group and <40 mg/day group, respectively. Likewise, the proportions of patients in the two treatment groups with stable disease and progressive disease following afatinib treatment were not significantly different. At the time of the data cutoff, 99 patients (67.8%) had discontinued afatinib. Among those patients, 34 had died, 58 had stopped at their physician’s discretion due to disease progression, and 7 had discontinued due to severe adverse effects. Forty patients (50.6%) from the 40 mg/day group received second-line treatment, with 27 patients of them receiving platinum doublet chemotherapy. Of the patients from the <40 mg/day group, 33 received second-line treatment, with 22 of them receiving platinum doublet chemotherapy (Table 3). The patients still on afatinib were censored for the TTF analysis. The TTF was 405 days for the 40 mg/day group versus 472 days for the <40 mg/day group (HR: 1.294, 95% CI: 0.8712-1.921, p=0.2271). The difference in the number of patients in the two groups who received palliative local radiotherapy applied to brain, bone, and lung lesions was not significant.

**Table 2:** Response of NSCLC patients to afatinib therapy

Characteristics	Total (n=146)	First-line treatment with afatinib		
		40 mg/day (n=79)	< 40 mg/day (n=67)	*p* value
Response to afatinib				0.8028 1.0000 0.9447 0.4152 0.7575
CR	3 (2.1)	2 (2.5)	1 (1.5)	
PR	102 (69.9)	55 (69.6)	47 (70.1)	
SD	30 (20.5)	15 (19.0)	15 (22.4)	
PD	11 (7.5)	7 (8.9)	4 (6.0)	
Time to treatment failure (TTF, median, days)	443	405	472	0.2271
Overall Survival (OS, median, days)	Undefined	Undefined	Undefined	0.8061

CR, complete response; PR, partial response; SD, stable disease; PD, progressive disease.

**Table 3:** Comparison of subsequent treatments after failure of first-line afatinib therapy

	First-line treatment with afatinib		
	40 mg/day (n=79, %)	<40 mg/day (n=67, %)	*p* value
Patient No.	40 (50.6)	33 (49.3)	1.0000
Doublet chemotherapy	27	22	0.8614
Cisplatin/Docetaxel	1	3	0.3333
Cisplatin/Paclitaxel Cisplatin/Vinorelbine	0 0	0 2	0.2089
Cisplatin/Gemcitabine	0	0	0.3649
Cisplatin/Pemetrexed	26	17	
Single agent treatment	13	11	1.0000
Docetaxel	2	2	1.0000 1.0000 1.0000 1.0000 1.0000 1.0000
Gemcitabine	0	0	
Vinorelbine	3	3	
Pemetrexed	0	0	
EGFR-TKI			
Erlotinib	1	0	
Gefitinib	3	3	
Osimertinib	4	3	
Subsequent treatment after second-line treatment	8	6	1.0000
Cisplatin plus pemetrexed	0	1	1.0000
Paclitaxel or docetaxel	2	1	1.0000
Vinorelbine	0	0	
Pemetrexed	0	0	
Gemcitabine	1	0	1.0000
EGFR-TKI (Erlotinib or Gefitinib)	5	4	1.0000

No., number.

The TTF values of the patients in each dosage regimen are shown in Figure 2. Kaplan-Meier analyses of the data indicated that the expected durations of survival were similar between the 40 mg/day treatment group and the <40 mg/day treatment group. The TTF values for the two groups were also similar for the patients in each group bearing the exon 19 deletion mutation or the L858R mutation in the EGFR in their tumors (for the exon 19 deletion, 364 days for the 40 mg/day group and 519 days for the <40 mg/day group, HR: 1.518, 95% CI: 0.8698–2.650, p=0.1045; for the L858R mutation, 447 days for the 40 mg/day group and 466 days for the <40 mg/day group, HR: 1.113, 95% CI: 0.5954–2.079, p=0.8343). In cases where the tumors harbored mutations other than exon 19 deletions and L858R mutations, with such other mutations typically being found at lower frequencies, the higher dose regimen resulted in a poorer duration of TTF than the <40 mg/day regimen. However, the total number of patients of this type in both treatment groups was very low, and hence this result needs further confirmation.

**Figure 1:**
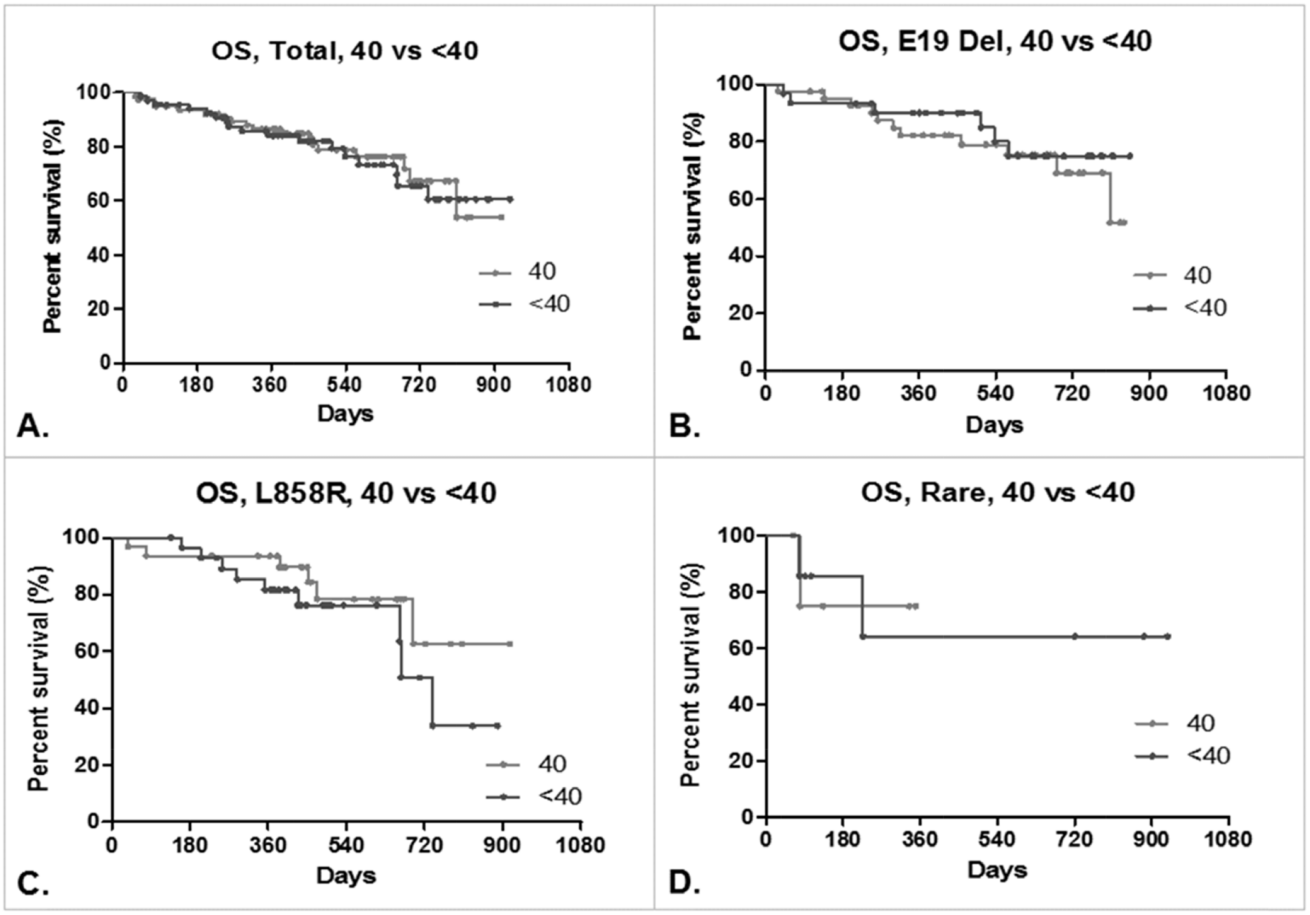
Overall survival (OS) of lung adenocarcinoma patients Kaplan-Meier curves of OS in patients treated with afatinib: **(A)** total, 40 mg/day (N=79) vs. <40 mg/day (N=67). Median: undefined vs. undefined days, HR: 0.9320, 95% CI: 0.4792 to 1.813, p=0.8061. **(B)** Patients bearing the exon 19 deletion mutation, 40 mg/day (N=42) vs. <40 mg/day (N=31). Median: undefined vs. undefined days, HR: 1.369, 95% CI: 0.5242 to 3.578, p=0.6135. **(C)** Patients bearing the L858R mutation, 40 mg/day (N=32) vs. <40 mg/day (N=29). Median: undefined vs. 738 days, HR: 0.5570, 95% CI: 0.2089 to 1.594, p=0.2889. **(D)** Patients bearing other rare mutations of the EGFR, 40 mg/day (N=5) vs. <40 mg/day (N=7). Median: undefined vs. undefined days, HR: 0.8695, 95% CI: 0.08265 to 9.148, p=0.9073.

**Figure 2:**
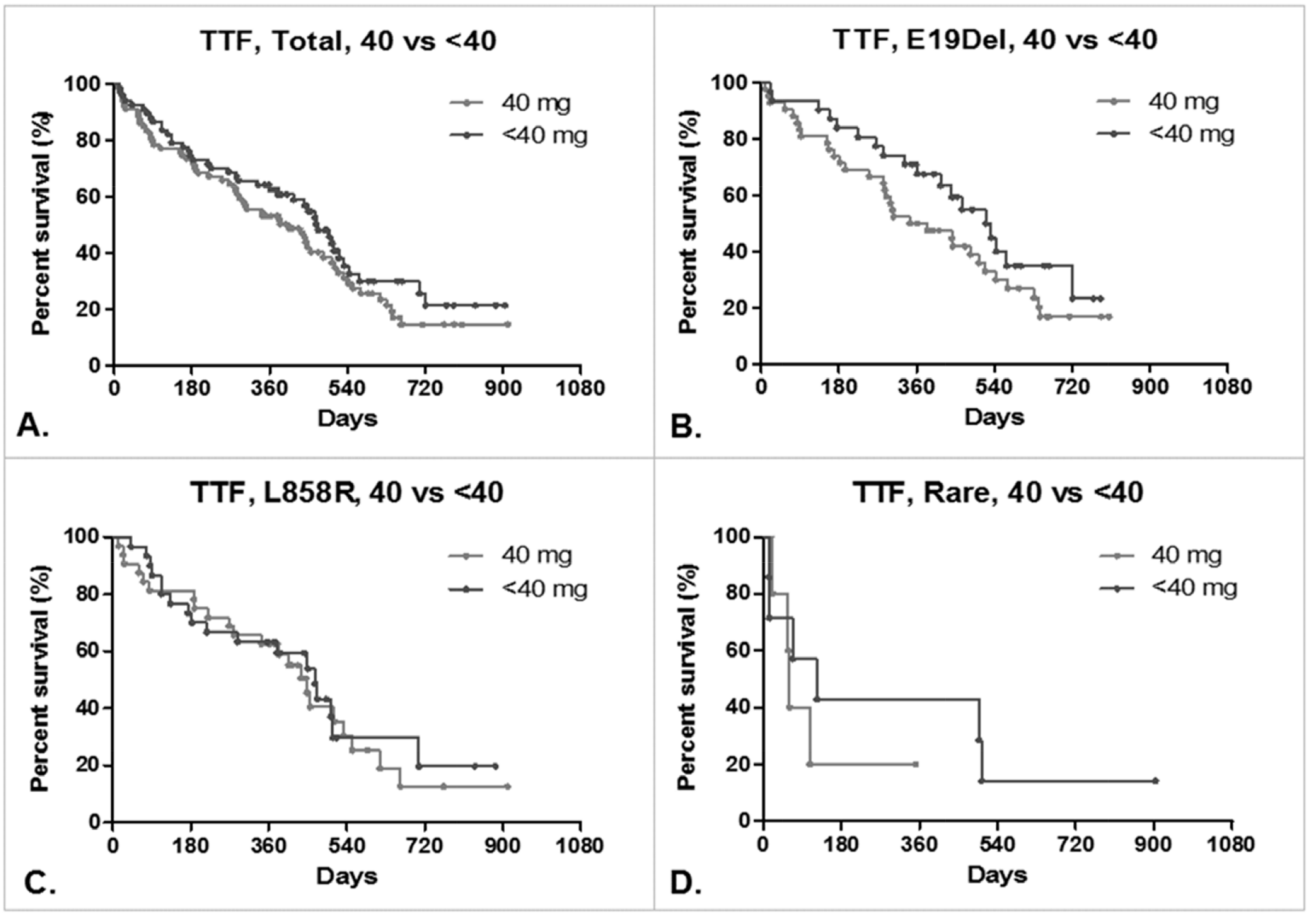
Time to treatment failure (TTF) of lung adenocarcinoma patients Kaplan-Meier curves of TTF in patients treated with afatinib: **(A)** total, 40 mg/day (N=79) vs. <40 mg/day (N=67). Median: 405 vs. 472 days, HR: 1.294, 95% CI: 0.8712 to 1.921, p=0.2271. **(B)** Patients bearing the exon 19 deletion mutation, 40 mg/day (N=42) vs. <40 mg/day (N=31). Median: 364 vs. 519 days, HR: 1.518, 95% CI: 0.8698 to 2.650, p = 0.1045. **(C)** Patients bearing the L858R mutation, 40 mg/day (N=32) vs. <40 mg/day (N=29). Median: 466 vs. 447 days, HR: 1.113, 95% CI: 0.5954 to 2.079, P=0.8343. **(D)** Patients bearing other rare mutations of the EGFR, 40 mg/day (N=5) vs. <40 mg/day (N=7). Median: 60 vs. 124 days, HR: 1.729, 95% CI: 0.4026 to 7.426, p=0.6207.

The rates of overall survival for patients bearing favorable EGFR mutations were similar in both treatment groups (Figure 1). The total number of patients with rare EGFR mutations in both groups was too low, however, to make any firm assessment about the relative responses to the different dosage levels in this regard.

The data used in this analysis was limited to the period from May 2014 to December 2015. Furthermore, in addition to the 34 patients who had died, 112 patients who were still alive at the cutoff date were censored. The overall survival rates of these patients are therefore not as complete an assessment as the TTF analysis, and further analyses are required to understand the impact of the reduced afatinib dosage on overall survival.

The frequency and severity of adverse events for both treatment groups were also recorded. Diarrhea, mucositis, paronychia, and skin rashes, predominantly in the Grade 0-2 categories, were the most common adverse events. The lower dose regimen did not result in a statistically significant reduction in the severity or frequency of adverse events, except anemia (Table 4).

**Table 4:** Comparison of adverse events due to the first-line afatinib therapy

Afatinib	40 mg/day, N=79 (%)				<40 mg/day, N=67 (%)				
Adverse event (Grade)	0	1	2	3-4	0	1	2	3-4	*p* value
Diarrhea	23 (29.1)	32 (40.5)	23 (29.1)	5 (6.3)	17 (25.4)	17 (25.4)	22 (32.8)	7 (10.4)	0.3666
Skin rashes	21 (26.6)	15 (19.0)	43 (54.4)	4 (5.1)	15 (22.4)	7 (10.4)	37 (55.2)	4 (6.0)	0.6480
Mucositis	50 (63.3)	10 (12.7)	21 (26.6)	2 (2.5)	30 (44.8)	8 (11.9)	20 (29.9)	5 (7.4)	0.2764
Paranychia	32 (40.5)	27 (34.3)	17 (21.5)	7 (8.7)	20 (29.9)	18 (26.8)	15 (22.4)	10 (14.9)	0.4696
Anemia	39 (49.4)	15 (19.0)	14 (17.7)	11 (13.9)	45 (67.2)	14 (20.9)	5 (7.4)	3 (4.5)	0.0390
Elevated GOT	48 (60.8)	27 (34.2)	3 (3.7)	1 (1.3)	52 (77.6)	14 (20.9)	1 (1.5)	0 (0)	0.1491
Elevated GPT	56 (70.9)	20 (25.3)	1 (1.3)	2 (2.5)	44 (65.7)	20 (29.9)	3 (4.5)	0 (0)	0.3237
Pneumonitis	77 (97.4)	1 (1.3)	1 (1.3)	0 (0)	66 (98.5)	1 (1.5)	0 (0)	0 (0)	0.6487

## DISCUSSION

EGFR mutations occur in greater frequency in individuals of Asian heritage, typically in lung adenocarcinomas [10]. The EGFR-TKIs gefitinib and erlotinib have been successfully utilized as first-line therapies for lung adenocarcinoma patients. The inherent issues with gefitinib and erlotinib, however, are that 1) both are reversible inhibitors and lack clinical activity in tumors harboring the EGFR T790M mutation [11] and 2) both can potentially result in drug-drug interactions, such that concurrent therapy with other drugs metabolized by cytochrome P-450-dependent enzymes and acid-reducing agents can interfere with the actions of gefitinib and erlotinib [12]. An irreversible ErB-family blocker, afatinib, retains its inhibitory effects on signal transduction and *in vitro* and *in vivo* cancer cell growth in tumors resistant to reversible EGFR inhibitors, such as those harboring the T790M mutation [13].

In this study, afatinib was administered as a first-line therapy to stage IIIB/IV lung adenocarcinoma patients harboring EGFR mutations. The results showed that this EGFR-TKI can serve as an effective first-line therapy for patients with metastatic lung adenocarcinomas, with 91.1% of the patients in the 40 mg/day dosage group showing a good disease control rate (Table 2, CR + PR + SD) in response to first-line afatinib therapy and 94.0% of the patients in the lower dosage group showing a good disease control rate in response to first-line afatinib therapy. These data indicate a lack of any statistically significant difference in the response to both afatinib dosage regimens.

Results from our study thus lend support to the hypothesis that EGFR-TKIs can be used as a first-line therapy for treatment of NSCLC patients. Reversible EGFR-TKIs like gefitinib and erlotinib have already been used for the treatment of lung cancer, but a comparative, controlled, and randomized trial of afatinib and gefitinib found that afatinib is more effective than gefitinib in treating NSCLC patients because it results in a greater duration of PFS (median 11.0 months versus median 10.9 months, HR: 0.73, *p*=0.017) and a longer time to treatment failure (median 13.7 months versus median 11.5 months, HR: 0.73, *p*=0.0073) [6]. Adverse events such as diarrhea and dermatological eruptions can sometimes be severe enough for patients to terminate afatinib therapy. Our results suggest, however, that the gradual adjustment of afatinib dosage, as per patient tolerance and physiological parameters like lean body mass, can be achieved. The results from this study show no significant differences in the pattern of disease progression for the different dosage levels with regard to the duration of TTF. Otherwise, the disease progression and subsequent therapy were similar in patients who received 40 mg/day and those who received a dose of < 40 mg/day.

For patients with the common EGFR mutations, the response to afatinib for both dosage regimens was also similar. The patient inclusion patterns (non-randomized) showed that the higher 40 mg/day dosage was perhaps not tolerated as well in general by women and by individuals with low body weight. These results are in agreement with the study published by Arrieta et al., which reported a correlation between tolerance of afatinib and low lean body mass [9]. In our study, a higher proportion of female patients (who typically have a less lean body mass) were included in the lower dosage regimen.

The results of this study show that afatinib dosages lower than 40 mg/day can be as effective as the 40 mg/day dose in terms of tumor response and TTF. At the time of data cutoff, the overall survival of the patients was not determined owing to the restricted period of data collection. Nonetheless, the results of this study provide a clear indication that dosages of afatinib can be calibrated as per the individual patient’s tolerance and experience of side effects.

Mutations of EGFR-TK, in addition to the exon 19 and exon 21 mutations, have been known to occur. However, since the incidence of these mutations in this study was low, no conclusive inferences can be drawn regarding the different dosage regimens for the patients bearing these mutations. Rather, additional studies are required in order to elucidate the use of afatinib as a first-line therapy in such patients.

A large percentage of the patients in each treatment group (69.6% in the 40 mg/day group and 70.1% in the <40 mg/day group) showed partial response after receiving first-line afatinib therapy. It would be interesting to know whether favorable EGFR-TK mutations persisted in the tumor tissue of these individuals or whether fresh mutations, with resultant resistance to afatinib therapy, emerged.

De-escalated dosages of afatinib showed a greater tendency towards longer periods of TTF in this study (Figure 2). These results are consistent with the results of other trials of afatinib therapy [6, 13, 14, 15]. The suspension of high-dosage afatinib therapy usually stems from the occurrence of higher grade diarrhea and cutaneous toxicity as adverse events. In one rare case, high cutaneous toxicity of afatinib was seen in a patient in whom the therapy was highly effective, to the extent that it resulted in the complete remission of the patient’s lung adenocarcinoma.

The severity and incidence of adverse events in this study were not statistically different in the treatment groups, except that the incidences of grade 2 and grade 3-4 anemia were higher in the 40 mg/day group. These findings also lend support to the notion that afatinib therapy can be calibrated to lower dosages based on assessments of the given patient’s response to the drug as well as other characteristics like cachexia or low lean body mass. Afatinib can be effective as a customized first-line therapy against NSCLC at dosages ranging from 30-40 mg/day. Our results are in agreement with recent post-hoc analyses of the LUX-LUNG 3 and LUX-LUNG 6 trials [16].

The necessity of treatment with chemotherapy drugs after afatinib therapy suggests that the residual tumor cells are most likely to be resistant to afatinib. A potential mechanism for the acquisition of resistance to afaitnib is the high persistence of the T790M mutation in lung cancer patients. Wu et al. reported the presence of this mutation in 47.6% of patients in a small clinical trial involving patients who were treatment-naive prior to afatinib exposure and patients who had been treated with other EGFR-TKIs prior to receiving afatinib treatment [17]. Other mutations in genes like *PIK3CA*, *HER2*, *KRAS*, *NRAS*, *MEK1*, and *AKT2* are not observed in cells that are resistant to afatinib. One possible reason for this is that afatinib is efficient at targeting cells that bear the exon 19 deletion and L858R mutations, with the result being that first-line afatinib treatment eliminates cells with these EGFR mutations. The remaining cells, a large proportion of which have T790M mutations, would therefore remain tumorigenic and metastatic. Post-hoc analyses of the mutation profiles of lung adenocarcinoma cells is likely to indicate new mechanisms of afatinib resistance. These investigations may also lead to the identification of drug targets that, when antagonized with the appropriate molecules, might lead to a therapy or therapies that complement afatinib in resolving lung adenocarcinomas.

## MATERIALS AND METHODS

### Patients

**Figure 3:**
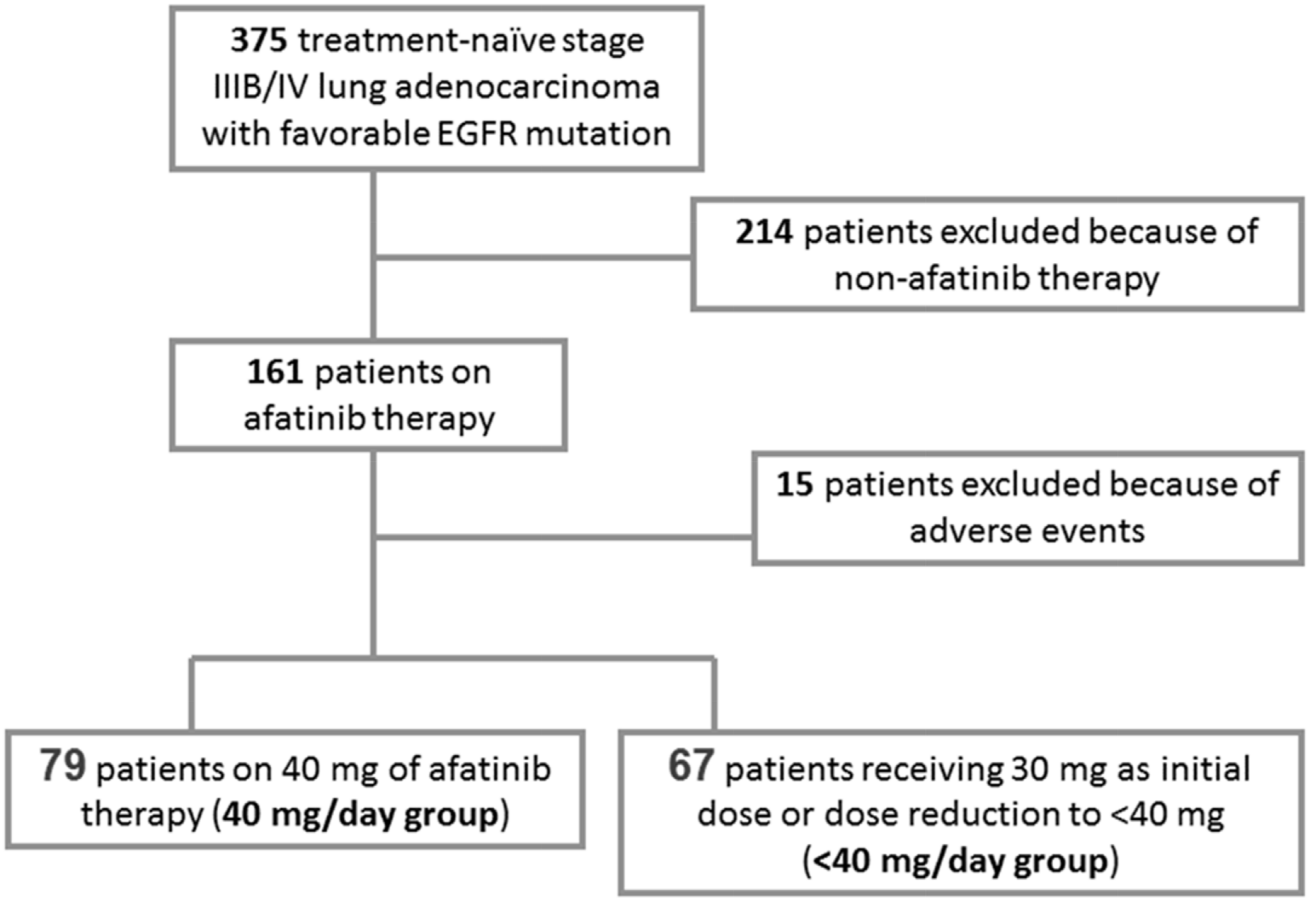
Schematic diagram of the inclusion/exclusion of patients in the study

This was a retrospective analysis. The data were retrieved from a prospectively registered patient database, and all the patients were followed up according to the lung cancer protocols of Chang Gung Memorial Hospital (CGMH, No.5, Fu-Hsin Rd, Kuai-Shan Dist, Taoyuan City 33333, Taiwan, R.O.C.). More specifically, stage IIIB/IV lung adenocarcinoma patients treated at CGMH between May 2014 (2014/05/05) and December 2015 (2015/12/14) were recruited for this study. The CGMH Institutional Review Board approved and authorized this study (IRB No.201601389B0), which was conducted according to the ethical principles of the Declaration of Helsinki, the International Council for Harmonisation Good Clinical Practice, and the prevailing national regulations and guidelines. A total of 375 treatment-naive patients were selected for initial consideration. All of these patients had mutations in the EGFR that are known to be sensitive to TKIs. 214 of the patients were subsequently excluded owing to the prescription of therapies other than afatinib, while 161 patients were treated with an afatinib regimen. A subset of 15 patients from this group was ultimately excluded due to their experience of severe adverse events resulting from their afatinib therapy, which caused the afatinib treatment to be halted within 30 days without any chest imaging of tumor progression being recorded. Of the remaining cohort of 146 patients, 79 patients were treated with a 40 mg/day dosage of afatinib throughout the treatment period or before the data collection cutoff date of 30 Nov 2016, while 67 patients were treated with a <40 mg/day dosage, with the patients in both groups receiving their doses orally in the form of a pill. In clinical practice, the decision to provide a dose of 40 mg/day or <40 mg/day of afatinib depended on the results of discussions between the patients and their physicians. Usually, if a patient expressed particular concern about the potential for diarrhea or skin-related side effects, the physician would prescribe an initial dose of <40 mg/day of afatinib (i.e., 30 mg/day). Alternatively, if the patient received 40 mg/day of afatinib initially and then later suffered from adverse events that were poorly tolerated, the physician would de-escalate the dose to <40 mg/day during the later period of treatment. More specifically, if the original dose of 40 mg/day was not tolerated, it was reduced by 10 mg/day by changing to a different strength tablet or adjusting the dosing interval. In the <40 mg/day group, 18 patients used 30 mg/day as a starting dose; subsequently, 2 of them escalated their dose to 40 mg/day based on their physician’s clinical judgment, and 1 of them down-titrated the dose to 20 mg/day. Forty-nine of the patients in the <40 mg/day group took 40 mg/day as their initial dose, and all of them subsequently reduced their dose to 30 mg/day due to side effects, after which only 2 of them required a further dose reduction to 20 mg/day. Figure 3 summarizes the process for the inclusion of patients in the study.

EGFR mutations were detected in patient biopsy samples using the direct sequencing and Amplified Refractory Mutation System (ARMS)-Scorpion methods, both of which have been well established in the Central Molecular Lab of the Department of Pathology of CGMH, a College of American Pathologists (CAP)-accredited laboratory. The best response was evaluated according to RECIST criteria (Response Evaluation Criteria in Solid Tumors, version 1.1). The adverse events were estimated according to CTCAE (Common Terminology Criteria for Adverse Events, version 4.03). The TTF was defined as the period from the first date of the afatinib therapy until the last date of the regimen. The TTF values for subjects who were still receiving the regimen without treatment failure at the time of the final follow-up date were treated as censored at the date of the last tumor assessment. Disease progression was defined as the development of any new site of disease on PET/CT and change in TNM stage (AJCC 7th ed.) were recorded [18]. Overall survival (OS) was defined as the period from the date of diagnosis until the date of death. For patients who were still alive at the time of the final follow-up date, survival was censored at the date of the last follow-up visit.

### Statistical analysis

Data are presented as means +/- standard deviation except where otherwise mentioned. For the data that did not approximate a Gaussian distribution, a nonparametric statistical analysis, the Mann-Whitney U-test, was performed for unpaired data to assess the significance of the difference between the two groups. Otherwise, the Student’s t-test was conducted for continuous variables to compare means between the two groups. Frequency distributions between the two groups were tested using the Chi-square or Fisher’s exact probability tests. Survival rates were calculated using the Kaplan-Meier method; a comparison of survival curves was then performed based on the log rank test, while HRs were determined via the Cox proportional hazards model. All the tests were two-sided and *p*<0.05 was considered statistically significant. GraphPad Prism (Version 5.0; GraphPad Software, San Diego, CA, USA) was used for all the statistical analyses.
